# Development and Validation of the Relational Needs Satisfaction Scale

**DOI:** 10.3389/fpsyg.2020.00901

**Published:** 2020-06-10

**Authors:** Gregor Žvelc, Karolina Jovanoska, Maša Žvelc

**Affiliations:** ^1^Department of Psychology, Faculty of Arts, University of Ljubljana, Ljubljana, Slovenia; ^2^Institute for Integrative Psychotherapy and Counselling, Ljubljana, Slovenia; ^3^Department of Psychology, Faculty of Mathematics, Natural Sciences and Information Technologies (UP FAMNIT), University of Primorska, Koper, Slovenia; ^4^Faculty of Education, University of Ljubljana, Ljubljana, Slovenia

**Keywords:** Relational Needs Satisfaction Scale, relational needs, attachment, relationship, psychotherapy, loneliness

## Abstract

The aim of the research was the development of a new scale for measuring the satisfaction of relational needs. In the first study, we developed 269 items based on Erskine’s description of eight relational needs. Five experts evaluated the items, and then they were pilot-tested on a sample of 221 participants. Using principal component analysis, we found five components related to five relational needs: authenticity, support and protection, having an impact, shared experience, and initiative from the other. In the second study, the Relational Needs Satisfaction Scale was tested on a sample of 255 participants and further refined with the help of factor analysis. The final version of the scale consists of 20 items and measures overall relational needs satisfaction and the five dimensions of relational needs. The reliability of the overall score was excellent, while subscales had acceptable to good reliability. The relational needs satisfaction positively and significantly correlates with the secure attachment style, self-compassion, higher satisfaction with life, and better well-being. In the third study, we confirmed both the five-factor model and the hierarchical model on the sample of 354 participants. We proposed that the hierarchical model is more congruent with the theoretical model, as all five dimensions of relational needs are aspects of one general dimension of relational needs satisfaction. The scale can be used in both psychotherapy and counseling and research related to different fields of psychology.

## Introduction

Richard Erskine developed a model of relational needs that is central in both relational integrative psychotherapy and contemporary transactional analysis (Erskine and Trautmann, 1996; Erskine et al., 1999; Erskine, 2015). Relational needs are the needs that are “unique to personal contact” (Erskine, 2015, p. 46) and can be satisfied only in a responsive human relationship. These needs are not only the needs of childhood but “are present throughout the entire life cycle from early infancy through old age” (Erskine, 2015, p. 47). The aim of the current research was the development of a new scale for measuring the satisfaction of relational needs.

The model of relational needs is based on attachment theory, object relations theory, transactional analysis, and self-psychology; these emphasize the need for relationships as the primary human motivation (Fairbairn, 1954; Berne, 1961, 1967; Bowlby, 1969; Kohut, 1971, 1977, 1984; Ainsworth et al., 1978; Stern, 1985; Fairbairn, 1986/1941; Winnicot, 1986/1960; Guntrip, 1992/1968). These theories have described the importance of a parent’s responsiveness to the relational needs of a child for healthy personality development. Bowlby (1969) describes that children’s need for attachment is a basic human need that is crucial for their protection from danger. An attachment system motivates infants to seek proximity and communication with caregivers. Therefore, attachment has an important evolutionary function – to heighten the possibility of the survival of the child. An attachment relationship is crucial for healthy personality development and development of the brain (Schore, 1994, 2001, 2003; Siegel, 1999, 2012; Cozolino, 2002).

Heinz Kohut’s self-psychology focuses on the importance of relationships for the maintenance of a cohesive self (Kohut, 1971, 1977). The child, for his/her psychological survival, needs an optimal empathic environment. Kohut (1984) described three main self-object needs in children: the need for mirroring, the need for idealization, and the need for twinship. He emphasized the importance of parental responsiveness to these needs for the development of a cohesive self.

There is increasing recognition that the early child-parent relationship influences brain development (Schore, 1994, 2001, 2003; Siegel, 1999, 2007; Cozolino, 2002). Schore (1994, 2003) has described the importance of affect regulation of the child’s psychobiological states for healthy brain development. Attunement and regulation of a child’s affects are crucial for establishing a sense of safety, connection, and secure attachment. Conversely, chronic misattunement, neglect, and abuse can have devastating consequences for a child’s personality and the brain’s development (Schore, 1994, 2001, 2003; Siegel, 1999). Attachment theory has described the importance of attachment needs through the life cycle (Hazan and Shaver, 1987; Bartholomew and Horowitz, 1991; Wallin, 2007). Responding to relational needs is thus important not only in childhood but throughout our whole life (Erskine et al., 1999; Erskine, 2015).

## A Model of Eight Relational Needs

Erskine et al. (1999) have described eight primary relational needs that were most often expressed by clients in psychotherapy:

(1) The need for security;
(2) The need to feel validated, affirmed, and significant within a relationship;
(3) The need to be accepted by a stable, dependable, and protective other person;
(4) The need for confirmation of personal experience;
(5) The need for self-definition;
(6) The need to have an impact on the other person;
(7) The need to have the other person initiate;
(8) The need to express love.

Erskine et al. (1999) describe *security* as the “visceral experience of having our physical and emotional vulnerabilities protected” (p. 124). The person also has a sense that he/she can be vulnerable and simultaneously in harmony with another. When this need is the foreground, the therapist’s task is to provide a sense of safety. The second need is *the need to feel validated, affirmed, and significant within a relationship.* In the psychotherapy relationship, the therapist may attune to this need by validating the significance and function of the client’s intrapsychic processes. The relational need for *acceptance by a stable, dependable, and protective other person* is the search for protection and guidance in a relationship. The therapist attunes to that need by being accepted by “a consistently, stable, dependable, and protective other person” (Erskine et al., 1999, p. 132). This need may be sometimes manifested in relationships as an idealization of another person. Erskine (2015) explains that idealization in psychotherapy or everyday life is not necessarily pathological, and could be understood as the need for intrapsychic protection. *The need for confirmation of personal experience* can be manifested through the desire to be in the “presence of someone who is similar, who understands because he or she has had a like experience, and whose shared experience is confirming” (Erskine, 2015, p. 50). If this need is in the foreground of psychotherapy, the therapist may share some of her/his experiences that are similar to the client. *Self-definition* is a relational need to be unique and different from others and to be respected and acknowledged for this uniqueness. It is the need to be different, which is in direct contrast to the need to be similar, which is expressed by the need for confirmation of personal experience. When this need is in the foreground of psychotherapy, the therapist consistently supports the expression of the client’s differences and individuality. *The need to have an impact* on the other person refers to “having an influence that affects the other in some desired way” (Erskine, 2015, p. 51). In a psychotherapeutic relationship, therapists are responsive to this need by being impacted by the client’s experience instead of being distant and uninvolved.

*The need to have the other person initiate* is the need to have another person initiate the exchange and make contact (Erskine et al., 1999). In a reciprocal relationship, both persons initiate contact and exchange. However, if the person is always in a position to initiate, the person may begin to doubt the investment of another person in the relationship. In a psychotherapeutic relationship, the therapist may respond to this need by providing initiation, direction, and guidance. *The need to express love* is “often expressed through quiet gratitude, thankfulness, giving affection, or doing something for the other person” (Erskine, 2015, p. 52). However, when an authentic need to express love is in the foreground, another person can accept the first person’s gratitude and acknowledge the need as part of the relationship. Erskine (2015) describes that the relational need to express love is often overlooked in psychotherapy and understood as manipulation, transference, or a violation of neutrality. However, when an authentic need to express love is in the foreground, the therapist can accept the client’s gratitude and acknowledge the need as part of the relationship.

There is currently no empirical research about the eight relational needs, however, several clinical observations and hypotheses regarding the nature of relational needs have been proposed (Erskine et al., 1999; Erskine, 2015). Erskine et al. (1999) argue that relational needs are dynamic; each relational need can become conscious as longing or desire, while the other relational needs are in the background. The authors also propose that an attuned and involved response by another person satisfies the relational need, and the need becomes less intense and goes into the background of awareness. When the person experiences that his/her relational needs are met in a relationship, the person experiences that he/she is being loved.

A model of relational needs may help psychotherapists develop a psychotherapy relationship that is attuned to the client’s emerging relational needs (Erskine, 2015). The therapist may react differently depending on what relational need is in the foreground. For example, when the need for confirmation of personal experience is in the foreground, a psychotherapist may share his/her personal experiences, whereas this would not be the optimal response if the need for protection would be in the foreground. The therapist responds empathically to the needs of the client and to the client’s painful recognition of past relational ruptures. While the psychotherapist cannot meet the client’s archaic needs, he/she can validate and normalize these needs, which may initiate a grieving process for the unsatisfied relational needs of the past. The concept of relational needs could be relevant in different psychotherapy approaches that give importance to the therapeutic relationship as a healing agent.

Erskine (2015) describes that relational needs are not only present in psychotherapy but are manifested in people’s everyday life. When relational needs are not met in a relationship, they may become more intense and upsetting. Erskine (2015) explains that the lack of satisfaction of relational needs can be “experienced as longing, emptiness, a nagging loneliness, or an intense urge often accompanied by nervousness” (p. 47). Continual non-satisfaction of relational needs can lead to frustration and anger in a relationship and can also gradually lead to loss of hope and meaning. Non-satisfaction of relational needs can also manifest in negative script beliefs about the self, others, and life, which are a cognitive defense against unsatisfied relational needs (Erskine and Moursund, 1988).

Erskine et al. (1999) proposed that the lack of satisfaction of relational needs may manifest in feelings of loneliness. In recent decades, there has been a lot of research showing the negative impact of loneliness and social isolation on mental and physical health. Loneliness has been associated with depression (Erzen and Çikrikci, 2018) and various other psychiatric disorders, such as alcohol abuse, child abuse, sleep problems, suicide, and Alzheimer’s disease (Mushtaq et al., 2014). Loneliness and social isolation is also a risk factor for mortality (Holt-Lunstad et al., 2015; Rico-Uribe et al., 2016) and is related with physical illnesses, such as diabetes, autoimmune disorders, cardiovascular diseases, cancer, and many others (Mushtaq et al., 2014). Loneliness is also negatively related to prosocial tendencies (Huang et al., 2016) and influences the mental and physical quality of life (Gerino et al., 2017).

Based on the mentioned research, we think that prolonged non-satisfaction of relational needs may have severe consequences for a person’s psychological and physical health. The concept of relational needs may provide a comprehensive understanding of the processes behind loneliness and unsatisfactory relationships and may be useful for developing interventions focused on preventing loneliness. Raising awareness of what are the unmet needs behind unsatisfactory relationships may provide a more precise understanding of relational difficulties in psychotherapy and preventative programs.

While the model of relational needs has roots in attachment theory, object relations theory, transactional analysis, and self-psychology, the model can also be related to other theories and research in human motivation. Erskine’s (2015) concept of the importance of relational needs satisfaction is congruent with the Baumeister and Leary (1995) description of the need to belong as a fundamental human motivation. Baumeister and Leary (1995) describe that “human beings have a pervasive drive to form and maintain at least a minimum quantity of lasting, positive, and significant interpersonal relationships” (p. 497). We propose that the eight relational needs refer to different aspects of this primary need for belongingness. Erskine’s (2015) model of relational needs also shares some similarities with the self-determination theory (Deci and Ryan, 2000), which proposes three primary human needs: autonomy, competence, and relatedness. The eight relational needs are in our opinion related to both autonomy and relatedness. Erskine’s (2015) need for self-definition is to some extent related to the need for autonomy, which is enhanced when other people “actively attempt to understand the person’s interests, preferences, and perspectives” (La Guardia and Patrick, 2008, p. 202). The other seven Erskine’s relational needs could be related to the need for relatedness, which is enhanced when other people “get involved with, show interest in, direct energy toward the person, and convey that the person is significant and cared for non-contingently” (La Guardia and Patrick, 2008, p. 202). We think that various Erskine’s (2015) relational needs provide a more nuanced understanding of this basic need for relatedness, which makes the concept useful in counseling and psychotherapy.

There already exist several instruments that measure constructs similar to Erskine’s (2015) concept of satisfaction of relational needs. Most instruments focus on general satisfaction in the relationship and not on separate relational needs. Hendrick (1988), for example, developed the 7-item “Relationship Assessment Scale” (RAS), which measures general relationship satisfaction in close relationships. The “Barret-Lennard Relationship Inventory” (BLRI) is another well-known instrument that is based on Carl Rogers’s (1957) theory of the necessary conditions of therapeutic change (Barrett-Lennard, 2015). It measures the level of regard, empathic understanding, and the unconditionality of regard and congruence (Barrett-Lennard, 2015). Positive and unconditional regard is in our opinion related to Erskine’s (2015) need for security and the need to feel validated, affirmed, and significant within a relationship. Another measure is the “Interpersonal Needs Questionnaire” (INQ), which measures thwarted belongingness and perceived burdensomeness based on the interpersonal theory of suicide (Van Orden et al., 2012). In terms of the Erskine’s model of relational needs, we think that the INQ measures the consequences of non-satisfaction of relational needs that may manifest in thwarted belongingness and perceived burdensomeness. The “Need to Belong Scale” (Leary et al., 2013) is another measure that shares similarities with the concept of relational needs. It is a 10-item scale that measures the strength of the need to belong based on the theory of Baumeister and Leary (1995).

Only a few existing measures focus on specific relational needs. The “Needs Satisfaction” is a 9-item measure based on the self-determination theory and measures satisfaction of the needs of autonomy, competence, and relatedness in a particular relationship (La Guardia et al., 2000). Banai et al. (2005) developed an instrument for measuring self-object needs according to Kohut’s (1971, 1977, 1984) theory, called the “Self Object Needs Inventory” (SONI). The inventory measures the constructs of mirroring, idealization and twinship, which partly correspond to Erskine’s needs for validation, acceptance and confirmation of personal experience. However, SONI measures the strength of the needs and not the satisfaction of relational needs in relationships.

Based on a review of different relationship measures, we conclude that there is a lack of measures that focus on the satisfaction of specific relational needs in relationships. Most measures focus on general relationship satisfaction or the strengths of the relationship needs. Also, there are currently no psychometrically valid instruments for measuring Erskine’s (2015) concept of satisfaction of relational needs. Because of that, we decided to develop a new scale for measuring the satisfaction of relational needs. The development of such an instrument would be important for the scientific validation and exploration of the concept of relational needs. It could be used in psychology research for measuring the satisfaction of relational needs in both non-clinical and clinical populations. The instrument could also be used in psychotherapy and counseling to assess the satisfaction of the client’s relational needs, and as a measure of the outcome of psychotherapy. It is expected that successful psychotherapy has an impact on the client’s relational capabilities, which may manifest in higher satisfaction of relational needs. Relational needs satisfaction is also a concept that could be important to assess in couples and family therapy. The instrument could also be used for empirical validation (or disconfirmation) of the model of relational needs.

The aim of our research was the development of the Relational Needs Satisfaction Scale (RNSS) based on Erskine’s (2015) model of eight primary relational needs. Three separate studies were conducted. In study 1, the aim was to develop the first item pool that would best describe the construct of relational needs and preliminarily test them on a pilot sample. In study 2, we further refined the scale and explored the validity and reliability of RNSS. In study 3, we confirmed the factor structure of the Relational Needs Satisfaction Scale. The research project adhered to the ethical standards for research of the Declaration of Helsinki revised in Fortaleza (World Medical Association, 2013), the ethical standards of the Slovenian Psychologist’s Association and the Slovenian Umbrella Association for Psychotherapy, and approved by the Institute for Integrative Psychotherapy and Counselling, Ljubljana.

## Study 1: Development of the Initial Pool of Items and the Pilot Study

The development of the scale was conducted in several phases. In the first phase, the aim was to develop the items that would best describe the relational needs construct. Relational needs were first described in a psychotherapy context; however, Erskine et al. (1999) see these needs as universal needs for human contact that can manifest in different relationships throughout life. In developing the RNSS, our goal was to develop items that would capture an individual’s general satisfaction of the relational needs in their life. We developed items that were not specific to any particular relationship. We created 269 items reflecting the eight dimensions of relational needs. Inspiration for the items was drawn from Erskine’s (2015) description of relational needs and clinical experience. The items were written from the perspective of the general satisfaction of relational needs in everyday life. The items were then evaluated in terms of their clarity and intelligibility of content. The items that were ambiguous, unclear, and that were too similar to each other were eliminated from the initial pool.

To evaluate the construct validity of items, five experts evaluated the items. The experts were all trained in integrative psychotherapy, were familiar with Erskine’s concept of relational needs, and used it in their everyday work with clients. Every expert evaluated each item and sorted it into an appropriate relational needs dimension based on literature and their clinical experience. The experts were also asked to provide feedback regarding the clarity and simplicity of the items and to provide suggestions for possible improvements. Items that were sorted into a “correct” dimension by at least four experts were put in the next phase of validation. After the selection of items in terms of content, 110 items were retained, which theoretically reflected the relational needs’ dimensions. The items described the satisfaction of a relational need for security (11 items), a need for validation (14 items), a need for acceptance (16 items), a need for confirmation of personal experience (11 items), a need for self-definition (11 items), a need to have an impact (18 items), a need to have others initiate (12), and, finally the need to express love (17 items). The items were randomly distributed in the first version of the Relational Needs Satisfaction Scale.

In the next phase of scale development, the aim was to preliminarily test the items on a community sample and select the items that would comply with both theoretical and psychometric criteria.

### Materials and Methods

#### Participants

The sample consisted of 221 participants from Slovenia, of which 181 (82%) of the participants were female, and 40 (18%) were men. Their ages ranged from 18 to 74 years (*M* = 35.9; *SD* = 12.7). Regarding their education, 34 (15%) completed primary school, 63 (28.5%) completed secondary school, 26 (11.86%) completed the 1st Bologna cycle or high school, 81 (36.6%) completed the 2nd Bologna cycle or had university degree, and 17 (7.7%) participants had completed a MsC or Ph.D. thesis. Participants were not compensated for their cooperation.

#### Instruments

We used the first form of RNSS, which was composed of 110 items reflecting the eight dimensions of relational needs. We gave some instructions to the participants: “Carefully read each statement and circle the correct answer. Use the scale below to make your choice. Please choose only one answer for each statement. There are no right or wrong answers. Be sure to answer every item.” Each item was rated on a 5-point Likert scale, from 1 (completely disagree) to 5 (completely agree).

#### Procedure

We conducted online research using site 1ka, which hosts online surveys in Slovenia. We shared the online survey consisting of RNSS and demographic questions through social media and e-mails. On the first page of the survey, we also described the general aims of the study and emphasized the voluntary participation and confidentiality of the answers. The administration took 10 min on average.

### Results and Discussion

We conducted a principal component analysis to investigate the internal structure and to reduce the number of items in the initial item pool. We considered a sample size to be big enough for the pilot study and the initial selection of items. This is congruent with Comrey and Lee (1992), who proposed that a sample size of 200 is a “fair” sample for conducting factor analysis. The value of Kaiser–Meyer–Olkin measure of sampling adequacy was 0.89, and Bartlett’s sphericity test showed statistical significance (χ^2^ = 16008.9, *p* < 0.000). These results suggested that the data were appropriate for principal component analysis. We first explored the eight-component solution since we theoretically predicted the eight main relational needs. However, the eight-component solution did not provide meaningful interpretation. Both the Scree test and Horn’s parallel analysis suggested five possible components. Five components accounted for 47.47% of the variance. We used varimax rotation, as we predicted the relatively independent dimensions of relational needs. Four components could be related to four main relational needs, as described in the theory: the needs for acceptance, for confirmation of personal experience, to have an impact, and the need for the initiative. The remaining component was saturated with four relational needs: the needs for security, for validation, for self-definition, and to express love. The results of the principal component analysis suggested that these relational needs are not independent dimensions but part of one dimension.

**FIGURE 1:**
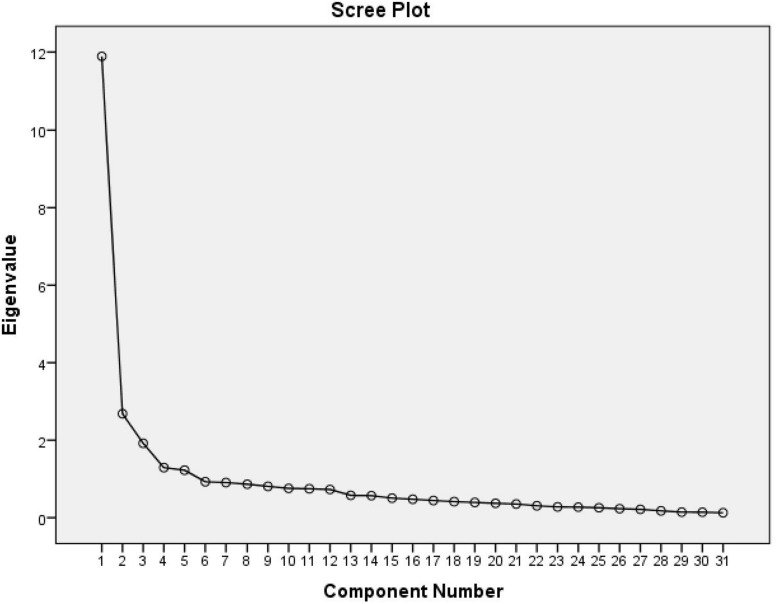
Scree plot of principal component analysis of the Relational Needs Satisfaction Scale.

The results showed that many items had low component loadings and saturated more than one component. In order to select the items for the final version of the scale, we excluded from further analysis all items that had a factor loading lower than 0.50. We also excluded all items that had loadings on more than one component. Reliability analysis was used to check the internal consistency of the scales and to select items that contributed to the lower reliability of the scale. We then again performed a principal factor analysis. This procedure was repeated several times in order to maximize the factor loadings of items on components and reduce items that were loading on more than one component. At the same time, we were also careful to retain items that were important in terms of the theoretical coverage of dimensions, even if they had factor loadings on more than one dimension. With the help of the aforementioned item selection, we retained 31 items that theoretically and psychometrically reflected the five dimensions of relational needs. We re-ran the principal component analysis. The Scree test proposed either a five-component or a one-component solution (see Figure 1).

The five-component solution was also supported by Kaiser’s criteria, as only five components had an eigenvalue > 1. Five components explained 61.33% of the variance. We used a varimax rotation as we predicted relatively independent relational needs dimensions. All items had loadings > 0.50 with the corresponding component and low loadings on other components (see Table 1). The first component explained 38.36% of the variance and consists of seven items that refer to Erskine’s need for acceptance by a stable, dependable, and protective other person. We named this scale, “Support and Protection.” When this need is met, the person feels at ease asking someone for help, protection, and support when in a place of distress. They can rely on someone stable, strong, and supportive. For example, note the item “*I have a strong, stable, and protective person in my life, whom I can rely on.*”

**TABLE 1:** Principal component analysis of Relational Needs Satisfaction Scale.

**N.**	**Content of the item**	**1**	**2**	**3**	**4**	**5**
89 (3)	I have a strong, stable, and protective person in my life whom I can rely on.	**0.68**	0.17	0.23	0.26	0.31
27 (3)	I am surrounded by reliable and strong individuals who are able to protect me.	**0.74**	0.26	0.11	0.19	0.17
12 (3)	In times of trouble, I have someone who stands by me and who is strong enough to handle my problems.	**0.75**	-0.02	0.02	0.20	0.24
4 (3)	I know a capable individual who would help me if I found myself in trouble.	**0.68**	0.15	0.14	0.00	-0.03
64 (3)	I have at least one person in my life who encourages me, protects me, or provides me with the information I need.	**0.74**	0.14	0.13	0.13	0.13
30 (3)	There is someone in my life I can rely on.	**0.77**	0.15	0.11	0.29	0.12
26 (3)	I do not know anyone that could protect me.	**0.70**	0.05	0.24	0.17	0.06
8 (6)	I have on occasions been told by people that I have influenced them or their decisions.	0.14	**0.70**	0.03	0.14	0.11
101 (6)	Other people often ask for my advice.	0.10	**0.69**	0.35	0.22	-0.05
35 (6)	Others often take my advice to heart.	0.13	**0.73**	0.18	0.05	0.14
66 (6)	I see that other people listen to my advice or my suggestions.	0.06	**0.63**	0.12	0.27	0.20
36 (6)	I feel that I have an influence on others.	0.13	**0.76**	0.23	0.13	0.11
29 (6)	I have noticed that other people sometimes follow my suggestions.	0.11	**0.70**	0.09	-0.16	0.22
103 (6)	Other people often ask about my opinion on a certain topic.	0.15	**0.64**	0.29	0.23	0.07
68 (4)	My social circle consists of people who share a similar life experience to me (e.g., a hobby, a profession, belonging to the same group or online forum).	0.03.	0.15	**0.70**	0.33	0.15
41 (4)	I have friends who I can share my experiences with as they too have experienced something similar.	0.27	0.25	**0.71**	0.02	0.10
53 (4)	I have contacts with people with similar interests.	0.09	0.23	**0.73**	0.28	0.27
34 (4)	I know people with a world-view similar to mine.	0.26	0.19	**0.64**	0.24	0.12
96 (4)	I know people who experience some things similarly to me.	0.15	0.30	**0.57**	0.19	0.42
79 (4)	There are people in my life with whom I share similar experiences.	0.28	0.25	**0.72**	0.14	0.28
102 (1)	I feel free to show my feelings to others and speak my mind because I know they accept me for who I am.	0.32	0.12	0.38	**0.56**	0.35
63 (1)	I do not have to pretend with people who are important to me.	0.36	0.15	0.22	**0.56**	0.26
77 (1)	I hardly have to hide anything in the company of people close to me.	0.34	0.05	0.21	**0.56**	0.35
109 (1)	I can show my true self to people who are important to me without fear of rejection.	0.32	0.16	0.36	**0.56**	0.14
7 (2)	I feel that people close to me understand me.	0.29	0.27	0.27	**0.52**	0.11
70 (5)	People encourage me to follow my own judgment regardless of their wishes.	0.30	0.24	0.08	**0.66**	0.09
72 (5)	When I get an idea that is different from most, other people important to me are quick to crush it.	0.03	0.04	0.14	**0.72**	0.16
108 (7)	Other people often help me even if I do not specifically ask them to.	0.12	0.13	0.23	0.16	**0.64**
14 (7)	Other people sometimes surprise me in a nice way.	0.19	0.17	0.13	0.09	**0.75**
65 (7)	People close to me would sometimes do things for me without me having to ask.	0.23	0.24	0.13	0.20	**0.58**
105 (7)	No one ever prepares a nice surprise for me.	0.08	0.10	0.21	0.22	**0.63**

*N = number of the item; Letters in the brackets refer to the Erskine’ original relational needs dimensions: (1) The need for security, (2) the need to feel validated, affirmed, and significant within a relationship, (3) the need to be accepted by a stable, dependable, and protective another person, (4) the need for confirmation of personal experience, (5) the need for self-definition, 6) the need to have an impact on the other person, and (7) the need to have the other initiate. Items with component loading > 0.50 are presented in bold.*

The second component explained 8.65% of the variance. It consists of seven items that correspond to the need to have an impact on another person. We shortly named this dimension as “Having an Impact.” This dimension refers to the need to feel that he/she has an impact on the other person. A person who has this need satisfied experiences that other people accept his/her opinion, advice, or ideas. She/he feels that she/he can affect other people and provoke a change in them. For example, note the item “*I feel that I have an influence on others.*”

The third component includes six items that correspond to Erskine’s (2015) need for confirmation of personal experience. The component explained 6.19% of the variance. We named this dimension “Shared Experience.” A person with this need met has people in his/her life with whom he/she can share similar interests and experiences. They have someone in life who experiences something similar and has some similar qualities. An example of the item “*There are people in my life with whom I share similar experiences.*”

The fourth component explained 4.17% of the variance and was saturated with seven items reflecting Erskine’s (2015) needs for security, validation, and self-definition. These relational needs were not found as separate dimensions, but were reflected in one overall dimension. We named and defined this dimension as the “Need for Authenticity.” The Need for Authenticity refers to the need of being authentic in a relationship with others. The person who has this need met, experiences that he/she can be with others what he/she truly is. It shows in the feeling of security, understanding, and respect by others. It also includes the feeling that other people accept the individual’s uniqueness or individuality. The satisfaction of the relational need for authenticity is therefore related to Erskine’s (2015) need for security, validation, and self-definition in a relationship. By feeling secure, validated, and being respected for his/her differences, the individual can be authentic with another person. An example of an item is “*I feel free to show my feelings to others and speak my mind because I know they accept me for who I am.*”

The fifth component explained 3.95% of variance and consists of four items that refer to the need to have another person initiate. The items describe the experience that other people sometimes surprise and help us without having to ask for it. It is about feeling that the other person does something for us without our request or demand. We named the scale as “Initiative from the Other.” For example, note the item: “*People close to me would sometimes do things for me without me having to ask.*”

Four components reflected the relational needs as described by Erskine et al. (1999): the need for acceptance by a stable, dependable, and protective other person, the need for confirmation of personal experience, the need to have an impact, and the need for initiative. Erskine’s (2015) relational need to express love was not found to be a separate dimension. Some of the items of this dimension saturated the Need for Authenticity dimension. However, factor loadings were lower than 0.40, so no items were retained in the final form of the scale.

The Scree test revealed that the one-factor solution could also be possible. The one-factor solution explained 36.40% of the variance. The eigenvalue of the factor was 11.89. The analysis of the factor matrix revealed that all items had satisfactory factor loadings (>0.40). The factor could be interpreted as an overall measure of the satisfaction of relational needs. It refers to the general experience of the person that his/her needs in a relationship are satisfied.

So, the results of the factor analysis of 31 items support the use of five scales measuring five main relational needs, and one scale reflecting the overall satisfaction of relational needs. There are five final scales: Authenticity, Support and Protection, Having an Impact, Shared Experience, Initiative from the Other, as well as Overall Score. The scales had acceptable to excellent reliability: 0.89 (Support and Protection), 0.86 (Having an Impact), 0.87 (Shared Experience), 0.86 (Authenticity), 0.73 (Initiative from the Other), and 0.94 (Overall Score).

## Study 2: Validity and Reliability of the Relational Needs Satisfaction Scale

The aims of Study 2 were to (1) explore the factor structure and the reliability of the Relational Needs Satisfaction Scale on an adult non-clinical sample and (2) examine the convergent validity of RNSS. In examining convergent validity, we focused on the correlation of RNSS with attachment, self-compassion, satisfaction with life, and well-being. We predicted that the satisfaction of relational needs is positively related to a secure attachment style and negatively to insecure attachment styles. People with a secure attachment feel secure and pleasant if they can rely on other people and vice versa (Bartholomew and Horowitz, 1991). They do not worry about being left alone or about acceptance from other people. As a result, we predicted that, compared to an insecure attachment, they may experience greater satisfaction of relational needs from other people. We also predicted that individuals who have their needs in relationships satisfied will experience greater satisfaction with life and well-being and that the satisfaction of relational needs is related to higher self-compassion. If the person feels that his/her relational needs are satisfied, we predicted that this could also be related to the internal relationship with himself/herself, which is manifested in greater self-compassion. We predicted that correlations with all described constructs would be low to moderate but positive and significant.

### Materials and Methods

#### Participants

Self-report questionnaires were administered to a sample of 255 participants from Slovenia, of whom 159 (62%) of the participants were female, and 96 (38%) were men. Their age ranged from 13 to 69 years with an average of 38.2 years (*SD* = 11). Regarding education, 65% of the participants had a university degree or higher education, 30% completed secondary school, and 4% fell into the “other” category. Regarding employment status, 77% of the sample were employed, 7% were unemployed, and 14% of the sample were high school or university students. Regarding relationship status, 34% were married, 41% were in a couples relationship, 20% were single, and 5% were divorced; two participants were widowed.

#### Instruments

##### Relational Needs Satisfaction Scale (RNSS).

The Relational Needs Satisfaction Scale consists of 31 items, reflecting five dimensions of relational needs (see Study 1). We also included additional 10 items, which were related to Erskine’s (2015) description of dimensions: The Need to Express Love, The Need for Security, The Need to Feel Validated, and The Need for Self-Definition. In study 1, these dimensions were not found as separate dimensions. By including these items, we wanted to make sure that items covered the whole theoretical construct of relational needs, even if they were not found significant in the pilot study. We gave participants instructions: “Carefully read each statement and circle the correct answer. Use the scale below to make your choice. Please choose only one answer for each statement. There are no right or wrong answers. Be sure to answer every item.” Each item was rated on a 5-point Likert scale, from 1 (completely disagree) to 5 (completely agree).

##### Self-Compassion Scale (SCS; Neff, 2003)

The Self-Compassion Scale is a self-report questionnaire that measures six components of self-compassion. It includes 26 items that measure three positive aspects of self-compassion (i.e., self-kindness, common humanity, and mindfulness) and three negative aspects (i.e., self-judgment, isolation, and over-identification). Each statement is rated on a 5-point Likert scale ranging from 1 (almost never) to 5 (almost always) (Neff, 2003). Subscale scores are calculated as means of subscale item responses. The total self-compassion score is computed as a mean of all six subscales. The Slovenian version of the SCS has acceptable validity and reliability (Uršič et al., 2019). A six-factor model and bifactor model of self-compassion were confirmed on the Slovenian sample. Reliability coefficients ranged from 0.66 to 0.84 for subscales and 0.91 for the total score (Uršič et al., 2019). We used the Self-Compassion Scale, as we predicted a positive relationship between the satisfaction of relational needs and self-compassion.

##### Satisfaction with life scale (SWLS; Diener et al., 1985)

The satisfaction with life scale includes five items, which measure global cognitive judgments of satisfaction with one’s life. Each statement is rated on a 7-point Likert scale ranging from 1 (strongly disagree) to 7 (strongly agree). Good psychometric properties of SWLS are reported. It has high internal consistency and test–retest reliability (α = 0.87) (Diener et al., 1985). In the Slovenian sample, the Cronbach’s alpha reliability coefficient was 0.87 (Uršič et al., 2019). Since relationship quality is related to higher satisfaction with life (Gustavson et al., 2016), we predicted a positive correlation between the satisfaction of relational needs and satisfaction with life.

##### Well-being Index (WHO-5; World Health Organization, 1998)

The WHO-5 is a short, self-administered questionnaire covering five positive items related to positive mood (relaxation), vitality (being active), and general interests (being interested in things). Each statement is rated on a 6-point Likert scale from 0 (not present) to 5 (constantly present). The WHO-5 has adequate validity in screening for depression and in measuring outcomes in clinical trials (Topp et al., 2015). In the Slovenian sample, Cronbach’s alpha reliability was 0.84 (Uršič et al., 2019). Since relationship dissatisfaction is related to depressive symptoms (Whitton and Kuryluk, 2012), we predicted that relational needs satisfaction would be related to higher well-being, measured with WHO-5.

##### Relationship Questionnaire (RQ; Bartholomew and Horowitz, 1991)

The RQ includes a description of four adult attachment styles: secure, preoccupied, fearful-avoidant, and dismissing-avoidant style. Each style is rated on a 7-point Likert scale ranging from 1 (disagree strongly) to 7 (agree strongly). Individuals with a secure attachment style feel pleasant and secure in a relationship with others and have a positive model of self and others. A preoccupied attachment style is related to a preoccupation with seeking emotional closeness to others and fears of being alone. A fearful-avoidant attachment style describes individuals with fears of closeness and intimacy and dismissing avoidant style individuals who feel well without close relationships and feel independent and self-sufficient. The Relationship questionnaire is a widely used measure to assess attachment styles. We predicted positive correlations between the satisfaction of relational needs and secure attachment and negative with insecure attachment styles.

#### Procedure

We conducted online research using site 1ka, which hosts online surveys. We created an online survey consisting of items related to relational needs and demographic questions. We also described the main goals of research and emphasized voluntary participation and the confidentiality of answers. The survey was shared through social media and e-mails.

### Results and Discussion

We performed a confirmatory factor analysis of the 31-item version of RNSS. The results of the confirmatory factor analysis showed unacceptable fit of the data to the model, with CFI and NNFI values below the level of acceptable fit (χ^2^ = 970.27, df = 424, CFI = 0.88, NNFI = 0.87, RMSEA {90% CI} = 0.07 (0.06, 0.08), SRMR = 0.06, AIC = 1939.46). Because of the unacceptable fit, we further refined the scale with the help of the principal axis factor analysis. We considered the sample size of 255 participants as adequate for factor analysis. This is congruent with rough guidelines of Comrey and Lee (1992), who proposed that a sample size of 200 is a “fair” sample and a sample size of 300 “good.” It is also congruent with Gorsuch (1983) and Streiner (1994), who suggested at least five participants per variable with at least 100 subjects.

We first conducted a principal axis factor analysis with an additional 10 items that we included to ensure full coverage of the construct of relational needs. The value of Bartlett’s test of sphericity was significant (χ^2^ = 5973.32, *p* < 0.000), and the Kaiser-Meyer-Olkin Measure of sampling adequacy was 0.92. Therefore, the data were suitable for factor analysis. Both the Scree test and Horn’s parallel analysis pointed to five factors solution explaining 49.54% of the variance. An analysis of the factor structure showed that none of the 10 additional items added to the relational needs construct and had factor loadings < 0.40.

We re-ran the principal axis factor analysis with 31 items selected in study 1. The Scree test proposed either a five-factor or one-factor solution. The five-factor solution was also supported by Kaiser’s criteria, as only five factors had an eigenvalue > 1. Five factors explained 56.38% of the variance. We used a varimax rotation, as we predicted the relatively independent relational needs dimensions. An analysis of the factor structure showed that some items had factor loadings < 0.50 and loading on more than one factor. When analyzing the content of the 31 items of RNSS, we found that some items were also similar to each other in terms of content and that the scale could be shortened. In the final version of the Relational Needs Satisfaction Scale, we retained four items from each scale, which showed that (a) had clear differentiation in terms of the content, (b) had the highest factor loadings on the corresponding factor, and (c) had the lowest cross-factor loadings.

We conducted the principal axis factor analysis of the 20 items version of the Relational Needs Satisfaction Scale (see Table 2). Both the Scree test and Kaiser’s criteria suggested a five-factor or one-factor solution. Five factors had eigenvalue > 1 and explained 69% of the variance. The mean level of communalities was 0.59, with most of the communalities higher than 0.50. All items had factor loadings > 0.50 on corresponding scales and low loadings on other scales. As factor structure was well determined with most factor loadings above 0.60, and communalities were above 0.50; this suggested that the sample size was big enough to obtain a factor solution that is stable (Guadagnoli and Velicer, 1988; MacCallum et al., 1999).

**TABLE 2:** Principal axis factor analysis of Relational Needs Satisfaction Scale.

**N.**	**Content of the item**	**1**	**2**	**3**	**4**	**5**
2 (A)	I hardly have to hide anything in the company of people close to me.	**0.75**	0.24	0.08	0.14	0.13
11 (A)	I feel free to show my feelings to others and speak my mind because I know they accept me for who I am.	**0.69**	0.17	0.17	0.26	0.24
12 (A)	I do not have to pretend with people who are important to me.	**0.77**	0.20	0.10	0.10	0.22
16 (A)	I can show my true self to people who are important to me without fear of rejection.	**0.83**	0.17	0.19	0.15	0.18
3 (P)	I have a strong, stable, and protective person in my life whom I can rely on.	0.15	**0.73**	-0.01	0.25	0.14
4 (P)	I know a capable individual who would help me if I found myself in trouble.	0.15	**0.60**	0.13	0.18	0.27
13 (P)	I have at least one person in my life who encourages me, protects me or provides me with the information I need.	0.28	**0.60**	0.13	0.13	0.27
17 (P)	In times of trouble, I have someone who stands by me and who is strong enough to handle my problems.	0.22	**0.77**	0.09	0.18	0.07
6 (H)	Others often take my advice to heart.	0.06	0.07	**0.68**	0.06	0.09
15 (H)	I feel that I have an influence on others.	0.15	0.03	**0.67**	0.04	0.21
19 (H)	I have noticed that other people sometimes follow my suggestions.	0.05	0.14	**0.78**	0.06	0.10
20 (H)	Other people often ask about my opinion on a certain topic.	0.20	0.01	**0.65**	0.23	0.16
7 (I)	Other people often help me even if I do not specifically ask them to.	0.27	0.18	0.09	**0.60**	0.11
9 (I)	Other people sometimes surprise me in a nice way.	0.08	0.18	0.16	**0.85**	0.11
10 (I)	People close to me would sometimes do things for me without me having to ask.	0.24	0.24	0.11	**0.50**	0.20
18 (I)	No-one ever prepares a nice surprise for me.	0.07	0.14	0.05	**0.68**	0.13
1 (S)	My social circle consists of people who share a similar life experience to me (e.g., a hobby, a profession, belonging to the same group or online forum).	0.11	0.08	0.18	0.29	**0.54**
8 (S)	I know people with a world-view similar to mine.	0.29	0.22	0.18	0.08	**0.58**
5 (S)	I know people who experience some things similarly to me.	0.15	0.31	0.22	0.12	**0.66**
14 (S)	There are people in my life with whom I share similar experiences.	0.38	0.20	0.19	0.20	**0.63**

*N, number of the item; Letters in the brackets refer to the names of the scales: A, Authenticity; P, Protection; H, Having an impact; I, Initiative from other; S, Shared experience; Items with component loading > 0.50 are presented in bold.*

We also explored a one-factor solution, which explained 37.24% of the variance and had an eigenvalue of 7.45. With the exception of one item (item 6), all items had factor loadings higher than 0.40, with most factor loadings > 0.50. The results indicate that the scale can also be used as a measure of overall relational needs satisfaction. The final version of the Relational Needs Scale includes 20 items, referring to the five main dimensions of relational needs and overall relational needs satisfaction (see Supplementary Table S1).

Table 3 shows descriptive statistics of the scales of RNSS. Subscales were calculated by adding the results on each scale and dividing the result by the number of subscale items. To compute a total relational needs score, we computed the results on all items and divided the result by the number of all items (20). The values of skewness and kurtosis were below the limits of -1 or 1 and did not reveal significant deviation from the normal distribution. The results show that all scales of the RNSS have α coefficients in the range from 0.81 to 0.90, which means that scales have good to excellent internal reliability. The highest reliability were for the Authenticity (0.90) and Total score (0.88) scales.

**TABLE 3:** Descriptive statistics and reliability of the Relational Needs Satisfaction Scale (*N* = 255).

**Dimension**	***M***	***SD***	**Min**	**Max**	**Skewness**	**Kurtosis**	**α**
Authenticity	3.96	0.79	1.00	5.00	-0.83	0.66	0.90
Protection	3.99	0.83	1.25	5.00	-0.72	-0.18	0.84
Having an impact	3.64	0.63	1.75	5.00	-0.07	0.01	0.81
Shared experience	4.01	0.69	1.50	5.00	-0.78	0.79	0.81
Initiative from other	3.02	0.46	1.00	4.00	-0.40	0.97	0.81
Total	3.72	0.50	2.00	4.75	-0.55	0.34	0.88

Table 4 shows that all scales of the RNSS significantly correlate between themselves at the 0.001 level. The strength of the correlation between main dimensions is in the range from 0.29 (weak) to 0.55 (moderate). Correlations between the main dimensions are theoretically expected, as the relational needs dimension overlap. Even though we have found significant correlations between the scales, the correlations are not higher than 0.55, which means that the scales measure distinct but related constructs. All dimensions of relational needs also strongly correlate with the overall relational needs satisfaction. This is theoretically expected, as all dimensions of relational needs describe different aspects of the concept of the relational needs’ satisfaction. This means that the general satisfaction of relational needs is related to the satisfaction of separate relational needs dimensions.

**TABLE 4:** Correlations between scales of the Relational Needs Satisfaction Scale (*N* = 255).

**Dimension**	**Authenticity**	**Protection**	**Having an impact**	**Shared experience**	**Initiative from other**	**Relational needs overall**
Authenticity	–					
Protection	0.50***	–				
Having an impact	0.34***	0.25***	–			
Shared experience	0.55***	0.51***	0.43***	–		
Initiative from other	0.43***	0.43***	0.29***	0.41***	–	
Total	0.80***	0.77***	0.61***	0.80***	0.65***	–

***Correlation is significant at the 0.001 level (2-tailed).

We also explored the convergent validity of the Relational Needs Satisfaction Scale, which in a predictable way, correlates with attachment styles, self-compassion, emotional well-being, and life satisfaction (see Table 5).

**TABLE 5:** Correlations between the dimensions of the Relational Needs Satisfaction Scale, attachment styles, self-compassion, well-being, and life satisfaction.

**Dimension**	**Authenticity**	**Protection**	**Having an impact**	**Shared experience**	**Initiative from other**	**Relational needs overall**
**Relationship Questionnaire**						
Securely attached	0.38***	0.304***	0.30***	0.32***	0.26***	0.45***
Fearful-avoidant	−0.36***	−0.24**	−0.18**	−0.25***	−0.16*	−0.33***
Preoccupied	−0.34***	−0.19**	−0.15*	−0.17**	–0.01	−0.25***
Dismissive-avoidant	–0.10	−0.17**	–0.10	−0.14*	–0.05	−0.16**
**Self-Compassion**						
Self-Kindness	0.25***	0.15*	0.29***	0.28***	0.09	0.29***
Self-Judgment	−0.15**	0.02	–0.09	–0.10	0.01	–0.09
Common Humanity	0.32***	0.23***	0.24***	0.33***	0.12*	0.35***
Isolation	−0.36***	−0.19**	−0.21**	−0.29***	–0.12	−0.33***
Mindfulness	0.29***	0.21**	0.29***	0.29***	0.08	0.33***
Over-identification	−0.19**	–0.07	−0.13*	−0.14*	–0.11	−0.18**
Overall self-compassion score	0.33***	0.17**	0.26***	0.29***	0.11	0.32***
**WHO5 Well-Being Index**	0.31***	0.19**	0.29***	0.31***	0.28***	0.37***
**Satisfaction with life scale**	0.38***	0.33***	0.33***	0.39***	0.27***	0.47***

***Correlation is significant at the 0.001 level (2-tailed). **Correlation is significant at the 0.01 level (2-tailed). *Correlation is significant at the 0.05 level (2-tailed).

As expected, we have found significant correlation between the satisfaction of relational needs and attachment styles. A secure attachment style is related to the greater satisfaction of all dimensions of relational needs. The strength of the correlation between an overall relational needs score and secure attachment is moderate (0.45). This finding is expected because, in a secure attachment style, people have positive working models of themselves and others and experience that other people can meet their needs and are available for contact in times of distress (Bartholomew and Horowitz, 1991). Among relational needs dimensions, the subscale Authenticity had the highest positive correlations with a secure attachment, which is logical as authenticity involves security and validation in a relationship, which are all significant components of a secure attachment style.

In contrast, we have found significant negative correlations between the satisfaction of relational needs and insecure attachment styles. A preoccupied attachment style is related to an experience that other people do not want to be as close to them as they would desire. Therefore, it is logical that they experience lower satisfaction of relational needs, as we have found in our research. We have also found a significantly low negative correlation between relational needs dimensions and the fearful-avoidant attachment style. People with such a style desire to have close relationships, but they have feelings of distrust and have difficulties in relying on other people. Because of the avoidance of relationships, they may experience lower satisfaction of relational needs, as we have found in our research. The correlation between the dismissive-avoidant attachment style and overall relational needs is significant, but very weak. The characteristics of the dismissive-avoidant attachment style is that they feel self-sufficient and independent, so they may deny the importance of relational needs, as is reflected in a very weak association with relational needs.

General relational needs satisfaction is positively and significantly related to higher self-compassion, specifically with higher self-kindness, common humanity, and mindfulness as well as lower isolation and over-identification. The results were theoretically expected and can be explained by the reciprocal relationship between self-compassion and the satisfaction of relational needs. Neff and Beretvas (2013) found that individuals with higher self-compassion show more positive relationship behavior (more care and support) than people who are less self-compassionate. Such a positive relationship behavior can influence other people who may, in turn, be more willing to respond by satisfying relational needs. Higher satisfaction of relational needs can also have a reciprocal influence on self-compassion. The person who feels that her/his relational needs are satisfied may also develop a more positive internal relationship, which is based on kindness and self-acceptance. If other people accept us for who we are, are supportive, can be impacted, and provide initiative, we can then also become more self-compassionate. This is in line with Rogers (1957), who proposed that self-acceptance is developed in relationships with others who provide empathy and unconditional self-regard.

Needs for authenticity, having an impact, and shared experience had the highest correlations with overall self-compassion. For meeting these needs, it is crucial a proactive behavior of the first person to get the satisfaction of his/her needs. That means that, to meet these needs, the person has to be willing to be authentic with another person, is proactive in making an impact, and is willing to share his/her world with another being. Self-compassion may be related to such proactive behavior, which leads to the satisfaction of relational needs. In contrast, the need for initiative does not correlate with self-compassion. The satisfaction of the need for initiation is more dependent on another person and does not necessarily involve the proactivity of the first person. Self-judgment significantly and negatively correlated only with the Authenticity dimension. Self-judgment may inhibit a person to express his/her real self in a relationship with others, which may be experienced as a lack of satisfaction of the need for authenticity.

We have also found significant positive correlations between all of the dimensions of relational needs and satisfaction with life and well-being. That means that greater satisfaction of relational needs is associated with greater satisfaction with life and emotional well-being. The results were expected, as we predicted that the satisfaction of relational needs is an essential ingredient of mental health and well-being. The results are congruent with research, which shows that relationship quality is related to greater satisfaction with life and well-being (Whitton and Kuryluk, 2012; Gustavson et al., 2016).

The results of the research show that the Relational Needs Satisfaction Scale has adequate internal validity, reliability, and convergent validity with measures of attachment styles, self-compassion, and satisfaction with life and well-being.

## Study 3: Confirmatory Factor Analysis of the Relational Needs Satisfaction Scale

In order to evaluate the validity of the RNSS on a new sample, we conducted research aimed to confirm the factor structure of the RNSS and investigate the reliability on a sample of young adults. The research was part of a larger project investigating relational needs satisfaction in young adults.

### Materials and Methods

#### Participants

The Relational Needs Satisfaction Scale (RNSS) was administered to a sample of 354 young adults from Slovenia, consisting of 248 female (70.1%) and 106 male (29.9%) participants. The age of the participants was between 18 and 30 years (*M* = 23.6; *SD* = 3). Regarding their education, 12 (3.3%) completed primary school, 149 (42%) completed secondary school, 126 (35.6%) completed the 1st Bologna cycle or high school, 63 (17.8%) completed the 2nd Bologna cycle, and 4 (1.1%) participants had completed a MsC or Ph.D. thesis. Participants were not compensated for their cooperation.

#### Instruments

##### Relational Needs Satisfaction Scale (RNSS)

The Relational Needs Satisfaction Scale consists of 20 items that refer to five main dimensions of relational needs (see Study 2). Instructions for participants were “Carefully read each statement and circle the correct answer. Please choose only one answer for each statement. There are no right or wrong answers. Be sure to answer every item.” The items are rated on a 5-point Likert scale from 1 (completely disagree) to 5 (completely agree).

#### Procedure

Data collection was carried out by two MSc students of psychology at the Department of Psychology, University of Ljubljana. Online research was conducted using the Slovenian online survey provider 1ka. The online survey link was shared via social networks using the snowball method – individuals were asked to share a survey among the acquaintances.

#### Psychometric Analysis

We used the Lisrel software version 10.20 to conduct a confirmatory factor analysis. We used the Maximum Likelihood Robust (MLR) estimation to estimate parameters, as the items did not have a normal distribution. The models were assessed with the following parameters of normed chi-square statistics (χ^2^/df), a comparative fit index (CFI), a non-normed fit index (NNFI), the root-mean-square error of approximation (RMSEA) with the accompanying 90% confidence interval (CI), a standardized root-mean-square residual (SRMR), and the Akaike information criterion (AIC).

The following criteria regarding the fit of the model were used. An acceptable fit is obtained when the CFI and NNFI values are 0.90 or higher, the RMSEA is 0.10 or lower. A normed chi-square lower than or equal to 3, and an SRMR of 0.10 or lower would also indicate an acceptable fit (Schermelleh-Engel et al., 2003). In order to compare different models between themselves, we also documented the Akaike’s information criterion (AIC). Models with a lower level of AIC show a better model fit.

### Results and Discussion

We performed a confirmatory factor analysis of the 20-item version of RNSS. We predicted that the sample size of 354 participants was adequate for confirmatory analysis, as the common rules of thumb suggest the sample size of at least 200, and 10 participants per variable (Myers et al., 2011).

Based on the theoretical model, we predicted that the separate relational needs dimensions refer to the general relational needs dimension. Therefore, the RNSS could measure not only the separate dimensions of relational needs, but also an overarching general factor. So, we tested both the 5-correlated factor model, the hierarchical model, and the uni-dimensional model of relational needs. The 5-factor model includes five distinct but correlated dimensions of relational needs. The hierarchical model predicts the overall relational needs factor, which is explained by five lower-order dimensions of relational needs. The uni-dimensional model predicts only one dimension of relational needs.

**FIGURE 2:**
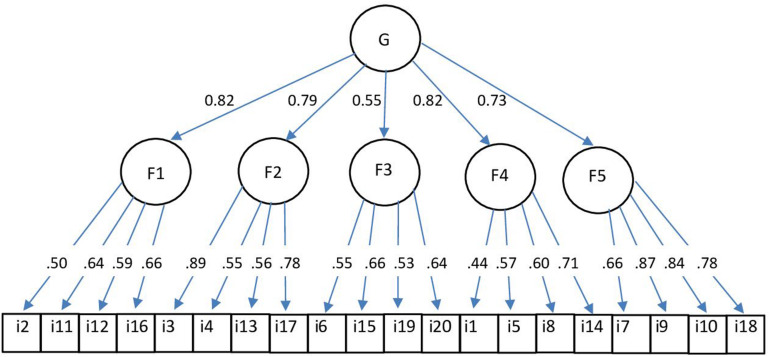
Hierarchical model of relational needs. G, the general factor of relational needs; Fl, Authenticity; F2, Support and Protection; F3, Having an Impact; F4, Shared experience; F5, Initiative from the Other.

Table 6 shows the results of the confirmatory factor analysis for RNSS. The results show that both the five-factor correlated model and the hierarchical model have a good fit. All indicators suggested a good fit to the theoretical model with CFI and NNFI indicators above 0.95. The uni-dimensional model did not fit the data well. The five-factor correlated model had a slightly better fit than the hierarchical model, however, we think that the hierarchical model is the most appropriate as it also accounts for the covariance among the first-order factors. The hierarchical model is also more congruent with theory, as all five dimensions of relational needs are aspects of one general dimension of relational needs satisfaction. The hierarchical model is presented in Figure 2. Relational needs dimensions had high factor loadings on the general factor (from 0.73 to 0.82), with the exception of the “Having an Impact” dimension, which had a factor loading 0.55. First-order factor loadings of items ranged from 0.44 to 0.89 and were all statistically significant (*p* < 0.001). The majority of items had factor loadings > 0.50, with the exception of item 1, which had the factor loading 0.44.

**TABLE 6:** CFA fit indices for the Relational Needs Satisfaction Scale.

	**χ ^2^**	***Df***	**χ ^2^/*df***	**CFI**	**NNFI**	**RMSEA {90% CI}**	**SRMR**	**AIC**
Model 1. Five factor correlated	241.55	160	1.51	0.97	0.97	0.05 (0.05, 0.06)	0.04	2725.17
Model 2. Hierarchical	250.56	165	1.58	0.97	0.96	0.06 (0.05, 0.06)	0.05	2741.96
Model 3. Unidimensional	846.70	170	4.98	0.78	0.75	0.13 (0.12, 0.14)	0.10	3552.74

*χ^2^, Satorra-Bentler Scaled Chi-Square (C3); CFI, comparative fit index; NNFI, non-normed fit index; RMSEA, root-mean-square error of approximation; CI, confidence interval; SRMR, standardized root mean square residual; AIC, Akaike’s information criterion.*

The internal consistency of the total score was excellent (α = 0.90). The reliability of relational needs dimensions was acceptable to good. Cronbach α for the subscales were: Authenticity (α = 0.80), Support and Protection (α = 0.85), Having an Impact (α = 0.81). Shared Experience (α = 0.73), and Initiative from Other (α = 0.83).

## General Discussion

The conducted research is the first empirical research regarding the Erskine et al. (1999) model of relational needs that is widely used in integrative psychotherapy and transactional analysis. Our research proposes five main relational needs comparing to eight relational needs described by Erskine et al. (1999). In the research, we could empirically confirm the four original Erskine et al. (1999) dimensions: the need for acceptance by a stable, dependable, and protective other person; the need for confirmation of personal experience; the need to have an impact; and the need for initiative. A new dimension, “Authenticity” was found, which includes the Erskine et al. (1999) relational needs for Security, Validation, and Self-Definition. These relational needs were not found to be separate dimensions. The results are not surprising, as relational needs for security, validation, and self-definition are theoretically interrelated. If we feel that we are validated in the relationship, we will also feel secure and experience that our individuality is respected. In contrast, if our need for self-definition is satisfied, we will also feel secure and validated. The Authenticity dimension, therefore, includes all three main relational needs. When a person feels secure, validated, and is respected for his/her uniqueness, the person will experience that he/she can be authentic in a relationship. The need for authenticity is to some extent similar to the need for autonomy in self-determination theory (La Guardia and Patrick, 2008), as it refers to the need that other people accept the person’s uniqueness and individuality without attempts to control and change.

The results also do not support Erskine’s (2015) relational need to express love as a separate dimension. Some of the items of this dimension saturated the Need for Authenticity dimension, however, factor loadings were low, so no items were retained in the final form of the scale. It may be that the need to express love is different from other relational needs, as it involves a person’s active engagement with other people – the desire to give as opposed to a desire to receive love. Other relational needs are more concerned with the satisfaction of the self-needs, whereas the need to give love is more oriented toward the benefit of other people.

The scale has adequate face validity and substantive-theoretical validity. Five experts selected the items and confirmed that items were appropriate to each relational needs satisfaction dimension (Study 1). The scale also shows adequate internal construct validity. In Study 2, we have found the clear five-factor structure of the instrument, with factors explaining 69% of the variance. The five-factor structure of relational needs was confirmed on a new sample of young adults (*N* = 354) (Study 3). The results of confirmatory factor analysis show that both the five-factor correlated model and the higher-order model had an acceptable fit, however, we proposed that the hierarchical model is the most appropriate because it accounts for the covariance among the first-order factors and is more congruent with the theory. The hierarchical model includes the general relational needs factor and five second-order dimensions of relational needs. The results indicate that the Relational Needs Satisfaction Scale can measure both overall satisfaction of relational needs and five dimensions of relational needs. The Relational Needs Satisfaction Scale has acceptable to good reliability for subscales and excellent reliability of overall score (0.90).

The Relational Needs Satisfaction Scale shows good convergent construct validity. The scale correlates predictably with attachment styles, self-compassion, well-being, and satisfaction with life. While the RNSS shows good convergent validity, the question remains if separate dimensions of relational needs show adequate discriminant validity. In further research, we propose an examination of both the convergent and discriminant validity of the subscales of RNSS.

The study has important implications for psychological practice. The Relational Needs Satisfaction Scale can be used for the assessment of relational needs in psychotherapy and counseling. Understanding which relational needs are unsatisfied may be important for treatment planning and evaluation of progress in counseling and psychotherapy. The scale can be used as an outcome measure in psychotherapy related to relational functioning. It may be particularly important for marital/couple counseling, as non-satisfaction of relational needs is often an underlying issue leading to problems in the couple relationship. The scale can be used in research related to interpersonal relationships in different fields of psychology, specifically for the understanding of the role of satisfaction of relational needs in loneliness, social isolation, and different clinical disorders.

### Limitations of the Research and Suggestions for Further Research

One of the limitations of the research was the characteristics of the samples. In both samples, the percentage of women was higher than men, and the samples consisted of people mainly with a university degree or secondary school education. The Relational Needs Scale should be tested in different samples, including clinical populations. Testing the scale in different countries could provide additional evidence of validity. The instrument can be used to assess the change of relational needs in different psychotherapy approaches, counseling, and preventative programs focused on loneliness and social isolation. The research could also investigate the role of relational needs satisfaction in different mental health disorders.

## Conclusion

The Relational Needs Scale is a new instrument for measuring the satisfaction of relational needs. It is based on the model of relational needs developed by Richard G. Erskine and colleagues at the Institute for Integrative Psychotherapy (Erskine and Trautmann, 1996; Erskine et al., 1999; Moursund and Erskine, 2004; Erskine, 2015) and has roots in attachment theory, object relations theory, self-psychology, and transactional analysis. The Relational Needs Scale includes 20 items referring to five dimensions of relational needs: Authenticity, Support and Protection, Having an Impact, Shared Experience, and Initiative from the Other. The scale also includes an overall score of relational needs satisfaction. The results of the confirmatory factor analysis show that both the five-factor model and the higher-order model of relational needs have an acceptable fit to the data. The Relational Needs Scale can be used as a measure of both five dimensions of relational needs and the general dimensions of relational needs. The Relational Needs Satisfaction Scale has acceptable to excellent internal reliability, and correlates in a predictable way with attachment styles, self-compassion, and satisfaction with life and well-being. It can be used for assessment of relational needs both in non-clinical and clinical settings and can be useful for research in different fields of psychology, including clinical, personality, and social psychology.

## Data Availability Statement

The datasets generated for this study are available on request to the corresponding author.

## Ethics Statement

The studies involving human participants were reviewed and approved by Institute for Integrative Psychotherapy and Counselling, Ljubljana, Slovenia. The patients/participants provided their written informed consent to participate in this study.

## Author Contributions

GŽ was responsible for the original conception, study design, data analysis, and major contribution to writing the manuscript. KJ played a role in item development, data collection, and writing of the manuscript. MŽ supervised the research and contributed to writing the manuscript.

## Conflict of Interest

The authors declare that the research was conducted in the absence of any commercial or financial relationships that could be construed as a potential conflict of interest.

## References

[B1] Ainsworth M. D. S. Blehar M. C. Waters E. Wall S. (1978). *Patterns of Attachment: A Psychological Study of the Strange Situation.* Hillsdale, NJ: Erlbaum.

[B2] Banai E. Mikulincer M. Shaver P. (2005). Selfobject” needs in Kohut’s self psychology: links with attachment, self-cohesion, affect regulation, and adjustment. *Psychoanal. Psychol.* 22 224–260. 10.1037/0736-9735.22.2.224

[B3] Barrett-Lennard G. T. (2015). *The Relationship Inventory: A Complete Resource and Guide.* West Sussex: Wiley-Blackwell.

[B4] Bartholomew K. Horowitz L. M. (1991). Attachment styles among young adults: a test of a four-category model. *J. Personal. Soc. Psychol.* 61 226–244. 10.1037//0022-3514.61.2.226 1920064

[B5] Baumeister R. F. Leary M. R. (1995). The need to belong: desire for interpersonal attachments asa fundamental human motivation. *Psychol. Bull.* 117 497–529. 10.1037/0033-2909.117.3.497 7777651

[B6] Berne E. (1961). *Transactional Analysis in Psychotherapy. A Systematic Individual and Social Psychiatry. A Systematic Individual and Social Psychiatry.* New York, Ny: Grove Press.

[B7] Berne E. (1967). *The Games people Play. The Psychology of Human Relationship.* New York, NY: Grove Press.

[B8] Bowlby J. (1969). *Attachment and Loss*, Vol. 1 London: Penguin Books.

[B9] Comrey A. Lee H. (1992). *A first Course in Factor Analysis.* Hillsdale, NJ: Erlbaum.

[B10] Cozolino L. (2002). *The Neuroscience of Psychotherapy. Building and Rebuilding the Human Brain.* New York, NY: W. W. Norton & Company.

[B11] Deci E. L. Ryan R. M. (2000). The “what” and “why” of goal pursuits: human needs and the self-determination of behavior. *Psychol. Inq.* 11 227–268. 10.1207/S15327965PLI1104_01 20204932

[B12] Diener E. Emmons R. A. Larsen R. J. Griffin S. (1985). The satisfaction with life scale. *J. Pers. Assess.* 49, 71–75. 10.1207/s15327752jpa4901_13 16367493

[B13] Erskine R. G. (2015). *Relational Patterns, Therapeutic Presence: Concepts and Practice of Integrative Psychotherapy.* London: Karnac Books.

[B14] Erskine R. G. Moursund J. P. (1988). *Integrative Psychotherapy in Action.* Newbury Park, CA: Sage Publication.

[B15] Erskine R. G. Moursund J. P. Trautmann R. L. (1999). *Beyond Empathy: A Therapy of Contact-in-Relationship.* Philadelphia: Brunner/Mazel.

[B16] Erskine R. G. Trautmann R. L. (1996). Methods of an integrative psychotherapy. *Trans. Anal. J.* 26 316–328. 10.1177/036215379602600410

[B17] Erzen E. Çikrikci Ö (2018). The effect of loneliness on depression: a meta-analysis. *In. J. Soc. Psychiatry* 64 427–435. 10.1177/0020764018776349 29792097

[B18] Fairbairn W. R. D. (1954). *Psychoanalytic Studies of the Personality.* New York, NY: Basic books.

[B19] Fairbairn W. R. D. (1986/1941). “A revised psychopathology of the psychoses and psychoneuroses,” in *Essential Papers on Object Relations*, ed. Buckley P. (New York, NY: New York University Press), 71–101.

[B20] Gerino E. Rollè L. Sechi C. Brustia P. (2017). Loneliness, resilience, mental health, and quality of life in old age: a structural equation model. *Front. Psychol.* 8:2003. 10.3389/fpsyg.2017.02003 29184526 PMC5694593

[B21] Gorsuch R. L. (1983). *Factor Analysis*, 2nd Edn Hillsdale, NJ: Erlbaum.

[B22] Guadagnoli E. Velicer W. F. (1988). Relation of sample size to the stability of componentpatterns. *Psychol. Bull.* 103 265–275. 10.1037/0033-2909.103.2.265 3363047

[B23] Guntrip H. (1992/1968). *Schizoid Phenomena, Object Relations and the Self.* London: Karnac books.

[B24] Gustavson K. Røysamb E. Borren I. Torvik F. A. Karevold E. (2016). Life satisfaction in close relationships: findings from a longitudinal study. *J. Happiness Stud.* 17 1293–1311. 10.1007/s10902-015-9643-7

[B25] Hazan C. Shaver P. (1987). Romantic love conceptualized as an attachment process. *J. Personal. Soc. Psychol.* 52 511–524. 10.1037//0022-3514.52.3.511 3572722

[B26] Hendrick S. S. (1988). A generic measure of relationship satisfaction. *J. Marriage Fam.* 50 93–98.

[B27] Holt-Lunstad J. Smith T. B. Baker M. Harris T. Stephenson D. (2015). Loneliness and social isolation as risk factors for mortality: a meta-analytic review. *Perspect. Psychol. Sci.* 10 227–237. 10.1177/1745691614568352 25910392

[B28] Huang H. Liu Y. Liu X. (2016). Does loneliness necessarily lead to a decrease in prosocial behavior? the roles of gender and situation. *Front. Psychol.* 7:1388. 10.3389/fpsyg.2016.01388 27695429 PMC5025448

[B29] Kohut H. (1971). *The Analysis of the Self.* New York, NY: International Universities Press.

[B30] Kohut H. (1977). *The Restoration of the Self.* Madison, CT: International Universities Press.

[B31] Kohut H. (1984). *How does Analysis Cure?.* Chicago, IL: University of Chicago Press.

[B32] La Guardia J. G. Patrick H. (2008). Self-determination theory as a fundamental theory of closerelationships. *Can. Psychol. Psychol. Can.* 49 201–209. 10.1037/a0012760

[B33] La Guardia J. G. Ryan R. M. Couchman C. E. Deci E. L. (2000). Within-person variation in security of attachment: a self-determination theory perspective on attachment, need fulfillment, and well-being. *J. Personal. Soc. Psychol.* 79 367–394. 10.1037//0022-3514.79.3.367 10981840

[B34] Leary M. R. Kelly K. M. Cottrell C. A. Schreindorfer L. S. (2013). Construct validityof the need to belong scale: mapping the nomological network. *J. Personal. Assess.* 95 610–624. 10.1080/00223891.2013.819511 23905716

[B35] MacCallum R. C. Widaman K. F. Zhang S. Hong S. (1999). Sample size in factor analysis. *Psychol. Methods* 4 84–99. 10.1037/1082-989X.4.1.84

[B36] Moursund J. P. Erskine R. G. (2004). *Integrative Psychotherapy: The Art and Science of Relationship.* Pacific Grove, CA: Thomson: Brooks/Cole.

[B37] Mushtaq R. Shoib S. Shah T. Mushtaq S. (2014). Relationship between loneliness, psychiatric disorders and physical health? A review on the psychological aspects of loneliness. *J. Clin. Diagnost. Res.* 8 WE01–WE04. 10.7860/JCDR/2014/10077.4828 25386507 PMC4225959

[B38] Myers N. D. Ahn S. Jin Y. (2011). Sample size and power estimates for a confirmatory factor analytic model in exercise and sport: a monte carlo approach. *Q. Exerc. Sport* 82 412–423. 10.1080/02701367.2011.10599773 21957699

[B39] Neff K. D. (2003). The development and validation of a scale to measure self-compassion. *Self Identity* 2, 223–250. 10.1080/15298860390209035

[B40] Neff K. D. Beretvas S. N. (2013). The role of self-compassion in romantic relationships. *Self Identity* 12 78–98. 10.1016/j.bodyim.2016.08.001 27597725

[B41] Rico-Uribe L. A. Caballero F. F. Martin-Maria N. Cabello M. Ayuso-Mateos J. L. Miret M. (2016). Association of loneliness with all-cause mortality: a meta-analysis. *PLoS One* 13:e0190033. 10.1371/journal.pone.0190033 29300743 PMC5754055

[B42] Rogers C. R. (1957). The necessary and sufficient conditions of therapeutic personality change. *J. Consult. Psychol.* 21 95–103. 10.1037/0033-3204.44.3.295 13416422

[B43] Schermelleh-Engel K. Moosbrugger H. Müller H. (2003). Evaluating the fit of structural equation models: test of significance and descriptive goodness-of-fit measures. *Methods Psychol. Res. Online* 8 23–74.

[B44] Schore A. N. (1994). *Affect Regulation and the Origin of the Self.* Hillsdale, NJ: Lawrence Erlbaum Associates.

[B45] Schore A. N. (2001). The effects of early relational trauma on right brain development, affect regulation, and infant mental health. *Infant Ment. Health J.* 22 201–269.

[B46] Schore A. N. (2003). *Affect Dysregulation & Disorders of the Self.* New York, NY: W. W. Norton & Company.

[B47] Siegel D. J. (1999). *The Developing Mind: Toward a Neurobiology of Interpersonal Experience.* New York, NY: The Guilford Press.

[B48] Siegel D. J. (2007). *The Mindful Brain. Reflection and Attunement in the Cultivation of Well Being*. New York: W.W. Norton & Company. 10.1176/appi.ajp.2007.07081292 22688157

[B49] Siegel D. J. (2012). *The Developing Mind: How Relationships and the Brain Interact to Shape Who We Are*, 2nd Edn. New York: The Guilford Press.

[B50] Stern D. (1985). *The Interpersonal World of the Infant. A View From Psychoanalysis and Developmental Psychology.* New York, NY: Basic Books.

[B51] Streiner D. L. (1994). Figuring out factors: the use and misuse of factor analysis. *Can. J. Psychiatry* 39 135–140. 10.1177/070674379403900303 8033017

[B52] Topp C. W. Østergaard S. D. Søndergaard S. Bech P. (2015). The WHO-5 well-being index: a systematic review of the literature. *Psychother. Psychosom*. 84, 167–176. 10.1159/000376585 25831962

[B53] Uršič N. Kocjančič D. Žvelc G. (2019). Psychometric properties of the Slovenian long and short version of the self-compassion scale. *Psihologija* 52 107–125. 10.2298/PSI180408029U

[B54] Van Orden K. A. Cukrowicz K. C. Witte T. K. Joiner T. E. Jr. (2012). Thwarted belongingness and perceived burdensomeness: construct validity and psychometric properties of the interpersonal needs questionnaire. *Psychol. Assess.* 24 197–215. 10.1037/a0025358 21928908 PMC3377972

[B55] Wallin D. J. (2007). *Attachment in Psychotherapy.* New York, NY: The Guilford Press.

[B56] Whitton S. W. Kuryluk A. D. (2012). Relationship satisfaction and depressive symptoms in emerging adults: cross-sectional associations and moderating effects of relationship characteristics. *J. Fam. Psychol.* 26 226–235. 10.1037/a0027267 22329388

[B57] Winnicot D. W. (1986/1960). “The Theory of the parent-infant relationship,” in *Essential Papers on Object Relations*, ed. Buckley P. (New York, NY: New York University Press), 233–254.

[B58] World Health Organization (1998). *Well-Being Measures in Primary Health Care/The DepCare Project*. Stockholm: WHO, Regional office for Europe.

[B59] World Medical Association (2013). World medical association declaration of helsinki: ethical principles for medical research involving human subjects. *JAMA* 310, 2191–2194. 10.1001/jama.2013.281053 24141714

