# Supplementary material for: Development and Validation of the Relational Needs Satisfaction Scale
Source: Front Psychol. 2020 Jun 10;11:901. doi: 10.3389/fpsyg.2020.00901 (PMC7298105; doi:10.3389/fpsyg.2020.00901)
Supplement: Supplementary file 1 [file Table_1.docx]

Supplementary Table S1

*Relational Needs Satisfaction Scale (RNSS)*

| N. | | Content of the item |
| --- | --- | --- |
| 1 | My social circle consists of people who share a similar life experience to me (e.g. a hobby, a profession, belonging to the same group or online forum). | |
| 2 | I hardly have to hide anything in the company of people close to me. | |
| 3 | I have a strong, stable and protective person in my life, whom I can rely on. | |
| 4 | I know a capable individual who would help me if I found myself in trouble. | |
| 5 | I know people who experience some things similarly to me. | |
| 6 | Others often take my advice to heart. | |
| 7 | Other people often help me even if I do not specifically ask them to. | |
| 8 | I know people with a world-view similar to mine. | |
| 9 | Other people sometimes surprise me in a nice way. | |
| 10 | People close to me would sometimes do things for me without me having to ask. | |
| 11 | I feel free to show my feelings to others and speak my mind, because I know they accept me for who I am. | |
| 12 | I do not have to pretend with people who are important to me. | |
| 13 | I have at least one person in my life who encourages me, protects me or provides me with the information I need. | |
| 14 | There are people in my life with whom I share similar experiences. | |
| 15 | I feel that I have an influence on others. | |
| 16 | I can show my true self to people who are important to me without fear of rejection. | |
| 17 | In times of trouble, I have someone who stands by me and who is strong  enough to handle my problems. | |
| 18 | No-one ever prepares a nice surprise for me. | |
| 19 | I have noticed that other people sometimes follow my suggestions. | |
| 20 | Other people often ask about my opinion on a certain topic. | |

*Note.*  N = number of the item.
